# Laparoscopic Transperitoneal Dismembered Pyeloplasty by Anderson-Hynes Procedure in Left Pelviureteric Junction Outlet Obstruction of a Horseshoe Kidney: A Case Report

**DOI:** 10.7759/cureus.117303

**Published:** 2026-10-01

**Authors:** Aman Arora, Ishaan Shinde, Irshad A Khan, Rishabh Singh Kalra, Arjun S Sandhu

**Affiliations:** 1 General Surgery, Military Hospital, Jamnagar, IND; 2 General Surgery, Seth GS Medical College and King Edward Memorial Hospital, Mumbai, IND; 3 Urology, Army Hospital (Research & Referral), New Delhi, IND; 4 Intern, M. S. Ramaiah Dental College and Hospital, Bengaluru, IND; 5 Urology, All India Institute of Medical Sciences, Jodhpur, IND

**Keywords:** anderson-hynes, case report, horseshoe kidney, laparoscopy, pelviureteric junction obstruction, pyeloplasty

## Abstract

Horseshoe kidney (HSK) is the most common renal fusion anomaly. The most common complication is Hydronephrosis, secondary to pelviureteric junction obstruction (PUJO). Less than 30 cases of minimally invasive reconstructive HSK procedures have been reported worldwide, starting in 1996. Unfamiliar caudal anatomy, aberrant lower pole vessels, and renal isthmus make laparoscopic pyeloplasty difficult. We report a case of PUJO in an HSK patient and a coexisting ventricular septal defect (VSD), who was managed with laparoscopic pyeloplasty.

A 23-year-old woman presented to the outpatient department with complaints of dull, aching pain in the left flank for the past year. There were no urinary complaints. There is a history of a VSD, which was surgically corrected at the age of 10. The patient was stable on examination, with no pallor, icterus, or edema. A midline sternotomy scar was seen. The abdomen was non-tender, with no organomegaly. The serum urea and creatinine were normal, and she had sterile urine. Abdominal ultrasound revealed an HSK. A renogram revealed left-sided hydronephrosis, with significant radiotracer retention in the pelvicalyceal system during delayed images, diagnostic of PUJO. A laparoscopic transperitoneal dismembered pyeloplasty was performed. The double-J stent was removed six weeks post surgery. A renogram performed 10 months after surgery showed mild hydronephrotic left moiety with a slow, non-obstructive clearance.

Despite being technically challenging owing to altered anatomy, laparoscopic pyeloplasty should be the treatment for HSK, with success rates comparable to open pyeloplasty and lesser post-operative morbidities.

## Introduction

Horseshoe kidney (HSK), or ren arcuatus, is a congenital anomaly caused by abnormal fusion of the inferior poles of both kidneys, occurring in approximately 0.25% of the population [1]. During fetal ascent from the pelvis, the fused kidneys become trapped beneath the inferior mesenteric artery and remain low in the abdomen, with the isthmus lying anterior to the great vessels. This condition is largely benign, but its altered anatomy predisposes to pelviureteric junction obstruction (PUJO), obstruction of urinary drainage at the junction between the renal pelvis and ureter. PUJO in HSK is more common than in normally positioned kidneys because of three anatomic features specific to HSK: aberrant or crossing lower-pole vessels that extrinsically compress the ureteropelvic junction, an abnormally high ureteral insertion into the renal pelvis, and malrotation of the kidney with the isthmus altering the normal course of the ureter [2]. These same features are what make laparoscopic reconstruction technically demanding, since the operating surgeon must identify and preserve aberrant vasculature while working around the isthmus in an unfamiliar caudal anatomic plane.

The Anderson-Hynes dismembered pyeloplasty, in which the stenotic ureteropelvic segment is excised, the redundant renal pelvis is trimmed, any aberrant vessels are transposed, and a tension-free ureteropelvic anastomosis is fashioned, is the standard reconstructive procedure for PUJO regardless of underlying renal anatomy [3]. In adults with HSK specifically, a laparoscopic transperitoneal adaptation of this technique has been reported in a small number of case series. The largest available adult series (15 patients) achieved a 93.3% long-term success rate with a low complication profile, comparable to outcomes in non-fused kidneys [4]. A systematic review of adult PUJO etiology further confirms that crossing or aberrant vessels are identified in approximately half of adult cases (50.5%), underscoring why vascular identification is a central technical concern in this population [5]. Of the two minimally invasive approaches available, a retroperitoneal approach minimizes gastrointestinal complications due to its extraperitoneal nature but offers a smaller working space and longer operative time [6], whereas a transperitoneal approach offers a wider working space and easier anastomosis [7], an advantage that is particularly relevant in HSK, where isthmus and vessel identification benefit from greater exposure.

This report aims to illustrate the technical considerations and perioperative outcome of transperitoneal Anderson-Hynes pyeloplasty in an adult with HSK and a coexisting, surgically corrected ventricular septal defect (VSD), highlighting how the anatomic and comorbidity-related factors in this patient informed our operative approach. Given that fewer than 30 minimally invasive HSK reconstructions have been reported worldwide since 1996 [4], and that most of the available literature is pediatric, we believe this adult case adds useful, condition-specific documentation of technique and outcome to a sparse evidence base.

## Case presentation

A 23-year-old female student of Indian ethnicity presented with a dull, aching pain in her left flank, first noticed approximately one year prior to presentation. The pain was intermittent, non-radiating, and had neither progressed to acute colic nor been associated with any clear exacerbating or relieving factor. She reported no urinary frequency, urgency, dysuria, hematuria, or febrile episodes, making an infective or calculous cause less likely at initial assessment; a musculoskeletal strain and chronic low-grade pyelonephritis were also considered as differentials before imaging redirected the workup toward an obstructive uropathy.

She had a history of a VSD surgically corrected 13 years earlier, with no known cardiac complications since. Her drug, family, and psychosocial histories were non-contributory. On examination, the abdomen was soft and non-tender with no palpable organomegaly; a well-healed midline sternotomy scar corresponding to her prior cardiac surgery was noted. She was afebrile, normotensive apart from a blood pressure of 136/86 mmHg, and had no pallor, icterus, cyanosis, clubbing, or lymphadenopathy. She was a non-smoker.

Laboratory investigations showed a blood urea nitrogen (BUN) of 34 mg/dL (reference range 7-20 mg/dL) and a serum creatinine of 0.9 mg/dL (reference range 0.6-1.1 mg/dL), summarized alongside her complete blood count in Table 1. The mildly elevated BUN, in the context of a normal serum creatinine, was interpreted as consistent with mild functional stress from chronic partial obstruction rather than significant global renal impairment; the preserved creatinine supported proceeding with elective, rather than urgent, surgical repair. Urine culture was sterile. Abdominal ultrasound, intravenous urography, contrast-enhanced CT/MRI (Figure 1), angiography, and a diethylenetriamine pentaacetic acid (DTPA) renogram were performed, together confirming an HSK with a hydronephrotic left moiety and significant radiotracer retention in the pelvicalyceal system on delayed imaging, diagnostic of PUJO.

**Table 1:** The patient's laboratory findings at the time of presentation BUN: Blood urea nitrogen

Parameter	Patient’s Values	Normal Range
Hemoglobin	12.6 g/dL	12-15.5 g/dL
Total Leukocyte Count	5,600/mm^3^	4,000-11,000/mm^3^
Differential Leukocyte Count	Neutrophils 70%, Lymphocytes 25%, Monocytes 3%, Eosinophils 2%, Basophils 0%	Neutrophils 40-60%, Lymphocytes 20-40%, Monocytes 2-8%, Eosinophils 1-6%, Basophils 0.5-1%
BUN	34 mg/dL	7-20 mg/dL
Serum Creatinine	0.9 mg/dL	0.6-1.1 mg/dL

**Figure 1:**
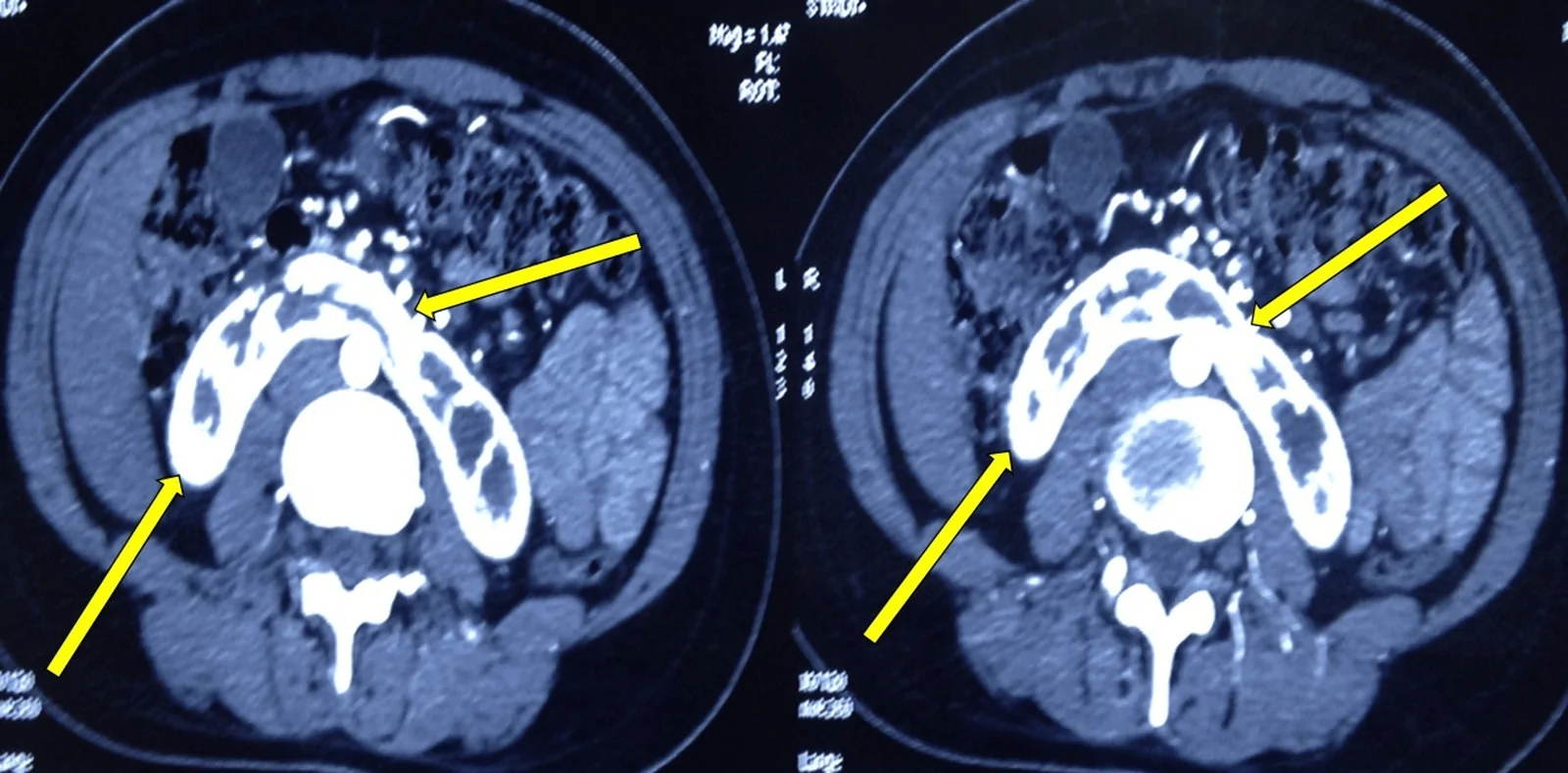
Contrast-enhanced axial CT demonstrating an HSK The arrows indicate the fused lower poles crossing the midline anterior to the great vessels (the renal isthmus), with associated dilatation of the left-sided pelvicalyceal system consistent with hydronephrosis secondary to pelviureteric junction obstruction. HSK: Horseshoe kidney

A laparoscopic transperitoneal dismembered pyeloplasty was planned following the Anderson-Hynes technique, in which the renal pelvis is transected circumferentially above the pelviureteric junction and the proximal ureter spatulated laterally to achieve a tension-free, water-tight repair [3]. The transperitoneal route was specifically chosen, rather than a retroperitoneal approach, to provide the wider working space needed to safely identify the isthmus and any aberrant lower-pole vessels before proceeding with dismemberment; port placement was adjusted slightly caudally and laterally relative to a standard (non-fused) kidney pyeloplasty to accommodate the lower, medially rotated position of the renal pelvis characteristic of HSK. No aberrant crossing vessel was identified intraoperatively in this patient, which we discuss further below in the context of the wider adult HSK literature.

The post-operative course was uneventful: the drain and sutures were removed on post-operative days 4 and 9, respectively, and the double-J stent was removed at six weeks. The patient was discharged without complications. Over 10 months of follow-up, she remained asymptomatic, with complete resolution of her presenting flank pain by the time of stent removal. Renal function was reassessed with a Tc-99m DTPA renogram, which showed a mildly hydronephrotic left moiety with slow, non-obstructive clearance, indicating preserved drainage without evidence of recurrent obstruction. The sequence of presentation and management is summarized in Figure 2.

**Figure 2:**
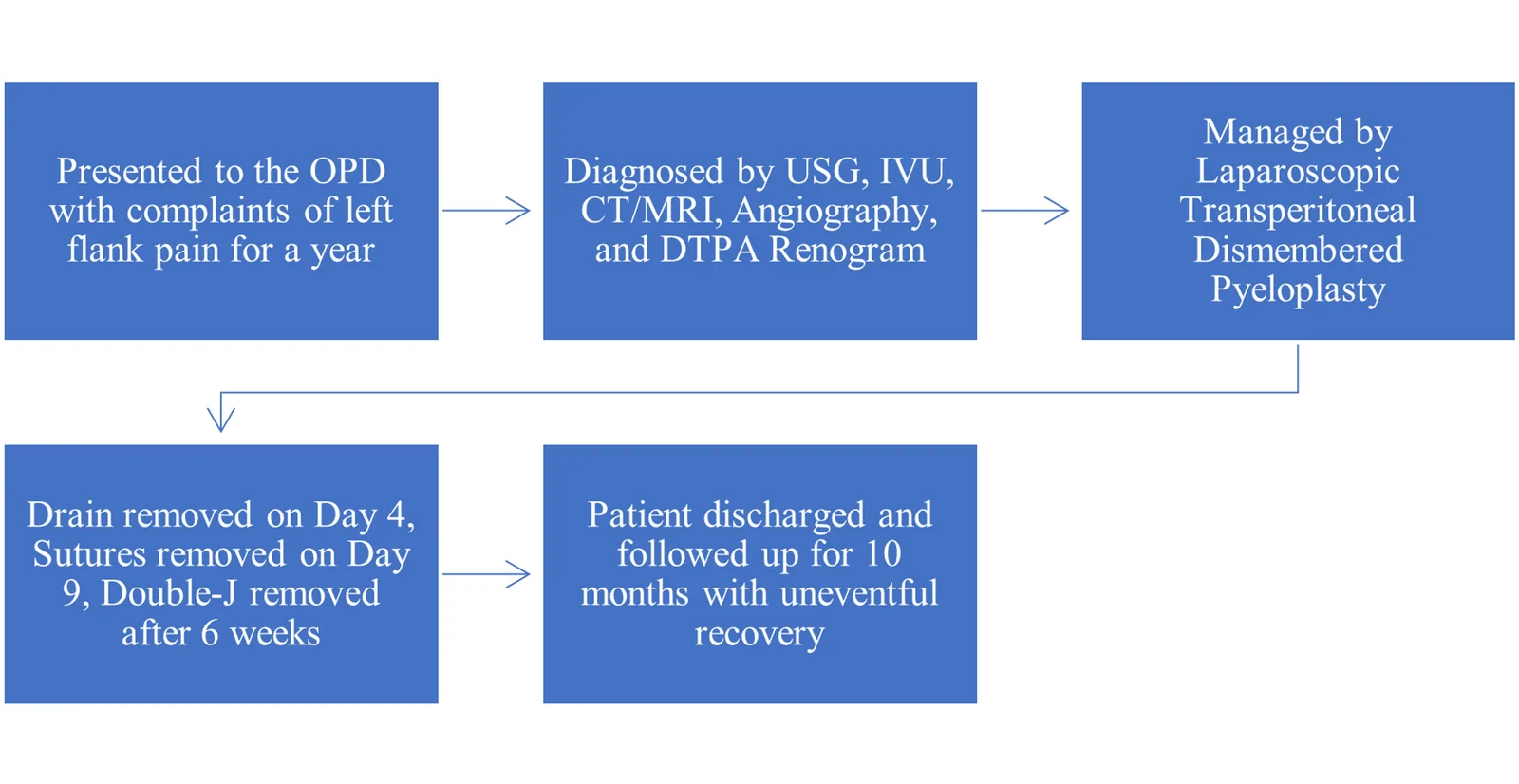
Sequence of events from the initial presentation to follow-up The timeline proceeds through five stages: (1) initial presentation with one-year history of left flank pain (day 0); (2) diagnostic workup comprising ultrasound, intravenous urography, CT/MRI, angiography, and DTPA renogram confirming HSK with PUJO; (3) laparoscopic transperitoneal Anderson-Hynes pyeloplasty; (4) post-operative recovery, with drain removal on day 4, suture removal on day 9, and double-J stent removal at six weeks, during which the presenting pain resolved completely; and (5) follow-up renogram at 10 months confirming non-obstructive clearance. DTPA: Diethylenetriamine pentaacetic acid; HSK: Horseshoe kidney; PUJO: Pelviureteric junction obstruction

## Discussion

HSK is the most common renal fusion anomaly, and hydronephrosis secondary to pelviureteric junction obstruction is its most frequent complication. The Anderson-Hynes procedure is the gold-standard reconstructive technique for PUJO irrespective of underlying renal anatomy, with reported success rates of 90-100% on long-term follow-up in the general population [8]. In HSK specifically, adult laparoscopic series have reported comparably favorable outcomes: a 15-patient transperitoneal laparoscopic series achieved a 93.3% success rate at extended follow-up [4], supporting the applicability of minimally invasive technique to this anatomically atypical population.

A key intraoperative consideration in HSK-PUJO is the presence of aberrant or crossing lower-pole vessels, which a systematic review of adult PUJO etiology found in roughly half of cases (50.5%) [5], and which frequently necessitate vessel transposition during dismembered pyeloplasty. In our patient, no such aberrant vessel was encountered, which places her case at the less complex end of the anatomic spectrum reported in the literature; we note this explicitly rather than implying that vessel transposition was required, since this distinguishes our operative findings from many published HSK-PUJO cases and may partly account for the straightforward post-operative course.

Regarding the recurring question of laparoscopic versus open pyeloplasty, a 2025 single-center comparative study in adults reported a laparoscopic success rate of 91.3%, together with shorter hospital stay and lower post-operative pain scores relative to open pyeloplasty [9], directly supporting our statement that the laparoscopic approach is comparable to open repair. We emphasize, however, that this comparative claim is drawn from the cited literature and not from our single-patient data; our case demonstrates that the technique was feasible and effective in this individual, but a single case cannot itself establish equivalence between approaches.

The coexistence of a surgically corrected VSD in our patient did not alter the operative plan, but it is a relevant perioperative consideration: pneumoperitoneum-associated hemodynamic and respiratory changes during laparoscopy warrant careful anesthetic monitoring in patients with a corrected cardiac anomaly, even when, as in this case, there is no residual cardiac dysfunction.

The value of this report lies in its detailed documentation of technique and short-term outcome in an adult HSK-PUJO patient, a population under-represented in a literature that remains predominantly pediatric. Its principal limitations, as in any single case report, are the absence of a within-study comparator arm against open pyeloplasty and a follow-up duration (10 months) too short to capture late recurrence of obstruction, which in the broader literature can occasionally manifest beyond one year.

## Conclusions

HSK is a common congenital renal anomaly frequently associated with pelviureteric junction obstruction. In this case, laparoscopic transperitoneal dismembered pyeloplasty by the Anderson-Hynes technique was technically feasible despite the altered anatomy of an HSK, and resulted in complete resolution of the patient's presenting symptom and preserved, non-obstructive renal drainage at 10-month follow-up. Consistent with a growing body of adult HSK-PUJO literature, this minimally invasive approach can be considered alongside open pyeloplasty in appropriately selected patients, offering shorter hospital stays, less post-operative pain, and favorable functional outcomes. However, broader conclusions regarding comparative equivalence with open surgery require larger case series or controlled studies with longer-term follow-up, which remains an acknowledged limitation of this single-patient report.
